# Risk factors for early neurological deterioration in acute isolated pontine infarction without any causative artery stenosis

**DOI:** 10.1186/s12883-022-02861-5

**Published:** 2022-09-03

**Authors:** Yinglin Liu, Hongmei Peng, Jian Wang, Lanying He, Jinghan Xu, Min Zheng, Yao Xu, Fan Xu

**Affiliations:** 1 Department of Neurology, Chengdu Second People’s Hospital, Chengdu, Sichuan 610011 People’s Republic of China; 2 Department of Neurology, Chengdu Qingbaijiang District People’s Hospital, Chengdu, Sichuan 610300 People’s Republic of China; 3 School of Medicine, University of Electronic Science and Technology of China, Chengdu, Sichuan 610041 People’s Republic of China; 4 Department of Radiology, Pingshan County People’s Hospital, Yibin, Sichuan 644000 People’s Republic of China; 5 Department of public Health, Chengdu Medical College, No. 601 Tianhui Street, JinniuDistrict, Chengdu, Sichuan 610500 People’s Republic of China

**Keywords:** Acute isolation pontine infarction, Risk predictors, Early neurological deterioration, Stroke subtypes, Infarct volume

## Abstract

**Background:**

This study aimed to investigate the risk predictors for early neurological deterioration (END) in isolated acute pontine infarction without any causative artery stenosis.

**Methods:**

In this retrospective study, patients with isolated acute pontine infarction within 72 h of symptom onset were enrolled between October 2017 and December 2021. END was defined as an increase in the National Institutes of Health Stroke Scale (NIHSS) score ≥ 2 points within the first week postadmission. Patients were divided into the END and the non-END groups. Multiple logistic regression analysis was used to evaluate independent predictors of END in patients with isolated acute pontine infarction.

**Results:**

A total of 153 patients were included in the final study (62 females; mean age, 67.27 ± 11.35 years), of whom 28.7% (47 of 153) experienced END. Multiple logistic regression analyses showed that infarct volume (adjusted odds ratio [aOR], 1.003; 95% CI, 1.001–1.005; P = 0.002) and basilar artery branch disease  (aOR, 3.388; 95% CI, 1.102–10.417; *P* = 0.033) were associated with END. The combined ROC analysis of the infarct volume and basilar artery branch disease for predicting END showed that the sensitivity and specificity were 80.9% and 72.6%, respectively.

**Conclusion:**

Basilar artery branch disease and infarct volume were associated with END in acute isolated pontine infarction and may be useful prognostic factors for neurological progression.

## Background

Early neurological deterioration (END) is relatively common in isolated acute pontine infarction (API), which is caused by small vessel disease or steno-occlusion of the orifice of a perforator at the parent artery. Based on previous studies, END in patients with isolated API has a high incidence of 25%–29% and is related to severe disability and poor outcomes [1–3]. However, the mechanism of END is currently unclear and may be related to hemodynamic factors, thrombus expansion, excitotoxicity and inflammation [4–6]

In previous studies [3, 7], END was reported to be related to the topographic location of the pontine infarction. However, another study showed that END was independent of the location and was not correlated with the size of the infarct [8]. Recently, the infarct size rather than topographic location of pontine infarction was suggested to be a possible predictor of END [9]. Similarly, these studies indicated that END was not related to severe stenosis of the basilar artery [3, 9, 10]. Therefore, the purpose of this study was to investigate the risk predictors for END in acute isolated pontine infarction without any causative artery stenosis.

## Methods

This retrospective study was approved by the Medical and Health Research Ethics Committee of the Second People’s Hospital of Chengdu (Chengdu, China) and adhered to the Declaration of Helsinki. Because it was a retrospective study, informed consent was not needed, and all included patient information was anonymous.

### Patient Selection

We retrospectively collected 153 patients with isolated API at Chengdu Second People's Hospital from October 2017 to December 2021. The inclusion criteria were as follows: (1) patients presenting within 72 h of onset; (2) diffusion-weighted imaging [DWI] within 48 h of admission showing isolated pontine infarction; and (3) patients with a modified Rankin scale (mRS) score ≤ 1 before admission. The exclusion criteria included (1) patients with pontine infarction with anterior circulation infarction or other vertebrobasilar infarction; (2) patients with vascular assessment (magnetic resonance angiography or computed tomography angiography) suggesting stenosis of the basilar artery (BA) (≥ 50%); (3) patients with cardiogenic embolism; (4) patients with severe cardiopulmonary, liver, or kidney insufficiency combined with malignant tumors; (5) patients with incomplete magnetic resonance imaging or poor imaging quality; and (6) patients with incomplete clinical data.

### Data collection

#### Demographic features and conventional risk factors

Two clinicians reviewed the electronic medical record system at Chengdu Second People’s Hospital to collect information, and a data extraction form was designed to record the patient information. Basic information included age, sex, hypertension, diabetes, smoking, drinking, and history of stroke, and the clinical data included time from onset to arrival, blood pressure at admission, baseline blood glucose level, National Institutes of Health Stroke Scale (NIHSS) score at admission, presence of END, NIHSS score at discharge, infarct site, treatment and hospital days. The following risk factors were evaluated: 1) hypertension: repeated blood pressure readings of ≥ 140/90 mmHg, a history of previous hypertension or use of antihypertensive drugs; 2) diabetes: a history of diabetes or the use of diabetes medications, or more than two measurements of fasting plasma glucose levels > 7.0 mmol/L or a random plasma glucose level > 11.1 mmol/L; 3) smoking: ≥ 10 cigarettes per day; and 4) drinking: alcohol consumption > 2 U/d [11]. Baseline examinations included routine laboratory tests, such as creatinine, alanine aminotransferase (ALT), aspartate aminotransferase (AST), triglycerides, total cholesterol, low-density lipoprotein (LDL), high-density lipoprotein (HDL), brain MRI, and computed tomography angiography (CTA)/magnetic resonance angiography (MRA) of the head.

### Brain mri protocol and analysis

**Fig. 1:**
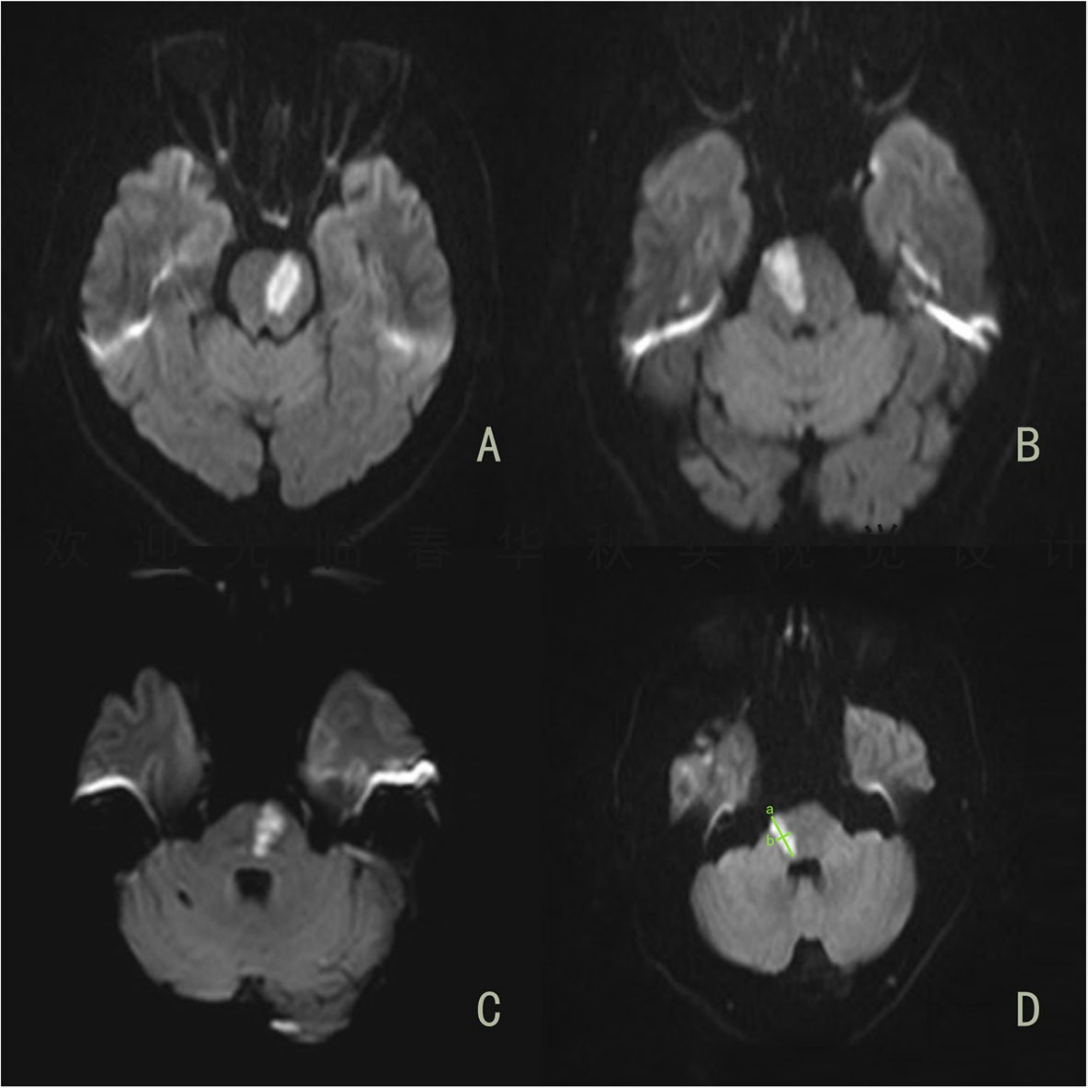
**(A**): upper pons; (**B**): middle pons; (**C**): lower pons; (**D**): API volume measurement based on DWI: a multiplied by b and then multiplied by the number of layers divided by 2

MRI was performed within 48 h of admission using a Siemens 1.5 T/3.0 T MRI scanner (Siemens AG, Munich, Germany). We recorded the location of the pontine infarct lesion, the volume, the diameter and width of the largest slice, and the distance of the lesion from the midline of the pons. The pons was divided into 3 sections based on the rostrocaudal location of the lesion by diffusion-weighted imaging (DWI; upper, middle, or lower pons) [3, 7]. The upper pons is characterized by a relatively round shape with a small, round-shaped aqueduct (Fig. 1A); the middle pons is characterized by its square-shaped fourth ventricle, large middle cerebellar peduncles, and silhouettes of trigeminal nerves (Fig. 1B); and the lower pons is characterized by a shape similar to that of the middle pons but with images of facial/acoustic nerves and grooves rather than trigeminal nerves (Fig. 1C). If more than one adjacent lesion was involved, the primary affected lesion was considered for grouping purposes. The diameter and width of the largest slice of each infarction were measured on DWI scans. We chose the diameter multiplied by the width of the largest infarction slice multiplied by the number of infarct slices multiplied by the slice thickness and then divided by two as the infarct volume (Fig. 1D). MRI scans were obtained at a 5-mm slice thickness.

### Stroke subtypes and END definition

**Fig. 2:**
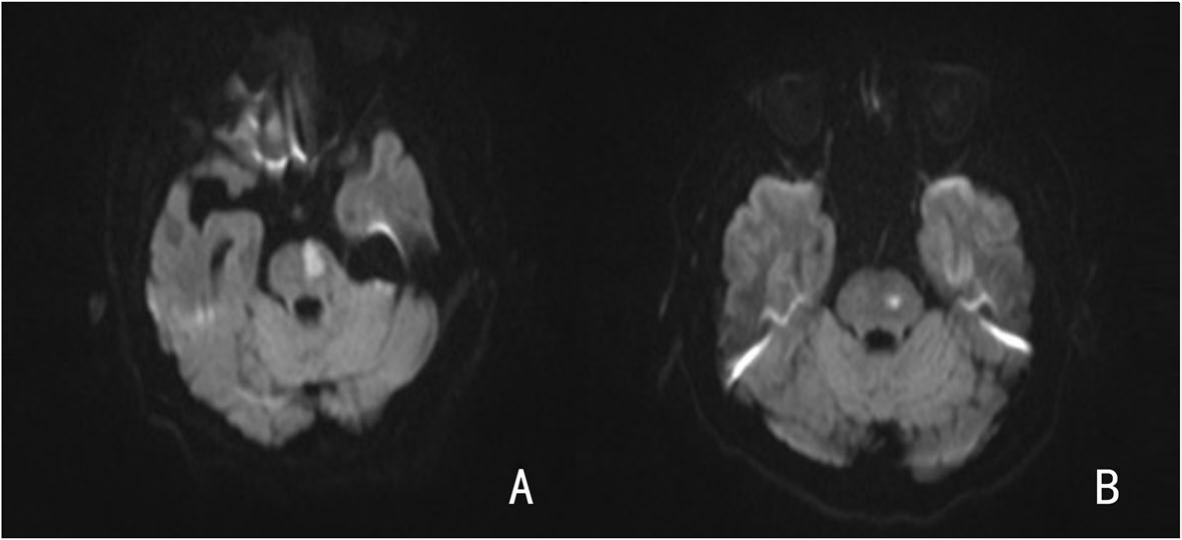
Representative MRI DWI images of patients with BABD (**A**) and SAD (**B**)

This retrospective study included patients withy 2 types of stroke: (1) basilar artery branch disease (BABD) characterized by an infarct that reached or approached the pontine surface without BA stenosis [12–14] (Fig. 2A) and (2) small artery disease (SAD) indicated by a deeper infarct without involvement of the ventral surface in the absence of BA stenosis [15] (Fig. 2B). END was defined as an increase in the NIHSS score ≥ 2 points within the first week after admission [13, 16].

### Statistical analysis

We used SPSS version 25.0 software (IBM Corp, Armonk, NY, USA) for statistical analysis. Continuous variables are expressed as the mean ± standard deviation (SD) or as the median and interquartile range (IQR). Differences between groups were compared using a t test or the rank-sum test. Categorical data are presented as frequencies (percentages), and the differences between groups were compared using the chi-squared test or Fisher’s exact test. Variables in univariate analyses (*P* < 0.10) were included in multivariate analysis. Receiver operating characteristic (ROC) analysis was used to assess the diagnostic value of our parameters for predicting END. Statistical significance was set at *P* < 0.05.

## Results

### Baseline characteristics

**Fig. 3:**
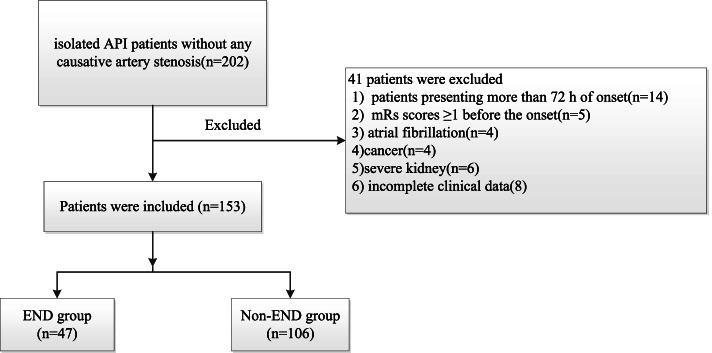
Flowchart of the selection of eligible subjects

A total of 202 patients with acute pontine infarctions were admitted to our neurology department from October 2017 to December 2021; 41 patients met the exclusion criteria, and eight patients had missing information. Finally, 153 patients with acute isolated pontine infarctions were included in the final study (91 males and 62 females; mean age, 67.27 ± 11.35 years)(Fig. 3). END occurred in 28.7% (47 of 153) of them after admission. The NIHSS score at discharge (*P* < 0.001) and number of hospital days (*P* = 0.007) were significantly higher in the END group than in the non-END group (Table 1).

**Table 1:** Clinical characteristics of patients with isolated API presenting with and without END

	END (*n* = 47)	Non-END (*n* = 106)	P
Age, mean ± SD, year	65.3 ± 11.9	68.1 ± 11.0	0.158
Female, sex, n (%)	18 (38.3)	44 (41.5)	0.709
Hypertension, n (%)	38 (80.9)	78 (73.6)	0.333
Diabetes, n (%)	22 (46.8)	36 (34.0)	0.131
Smoking, n (%)	12 (25.5)	20 (18.9)	0.350
Drinking, n (%)	5 (10.6)	17 (16.0)	0.380
History of ischemic stroke, n (%)	0 (0.0)	2 (1.9)	0.343
Blood pressure at admission			
SBP, mean ± SD, mmHg	151.5 ± 19.9	152.2 ± 24.3	0.87
DBP, mean ± SD, mmHg	82.7 ± 12.3	85.5 ± 13.9	0.234
Baseline blood glucose level, median (IQR), mmol/L	7.2 6.2, 11.2)	6.7 (5.5, 11.2)	0.260
Arrival time, median (IQR), hours	20.0 (7.0, 24.0)	24.0 (10.0, 48.0)	0.085
NIHSS score at admission, median, mean ± SD	3.9 ± 2.1	3.9 ± 2.6	0.992
NIHSS score at discharge, median (IQR)	4.9 ± 2.5	2.4 ± 2.0	0.000
Number of hospital days	13.2 ± 3.1	11.8 ± 3.0	0.007
Laboratory tests			
Total cholesterol, mmol/l, mean ± SD	4.7 ± 1.4	4.7 ± 1.3	0.958
Triglycerides, mmol/l, median (IQR)	1.47 (1.05,2.12)	1.60 (1.05, 2.31)	0.826
HDL, mmol/l, median (IQR)	1.11 (0.93, 1.26)	1.18 (1.01, 1.36)	0.062
LDL, mmol/l, mean ± SD	2.9 ± 1.2	2.7 ± 1.0	0.490
Creatinine, mmol/l, mean ± SD	66.6 ± 15.7	72.3 ± 23.3	0.08
AST, mmol/l, median (IQR)	19.0 (15.0, 24.0)	19.0 (15.0, 25.0)	0.960
ALT, mmol/l, median (IQR)	22.0 (17.0, 31.0)	20.0 (16.0, 27.2)	0.197
Treatment, n (%) 0.168			
IVT + DAPT	7 (14.9)	6 (5.7)	
DAPT	32 (68.1)	80 (75.5)	
Anticoagulation	8 (17.0)	20 (18.9)	

*SD* Standard deviation, *SBP* Systolic blood pressure, *DBP* Diastolic blood pressure, *IQR* Interquartile range, *NIHSS* National institutes of health stroke scale, *HDL* High-density lipoprotein, *LDL* Low-density lipoprotein, *AST* Aspartate aminotransferase, *ALT* Alanine aminotransferase, *IVT*, Intravenous thrombolytic therapy, *DAPT* Dual antiplatelet therapy

There were no significant differences in sex, age, hypertension, diabetes, drinking, smoking, baseline blood glucose level, history of ischemic stroke, blood pressure at admission, laboratory results, initial NIHSS score, or treatment after admission between the 2 groups (*P* > 0.05; Table 1).

Of the 153 patients, 86 patients (56.2%) had BABD, and 67 patients (43.8%) had SAD. The proportion of patients with END within one week after admission was 44.2% (38 of 86) in the BABD group and 13.4% (9 of 67) in the SVD group.

DWI indicated that upper pontine infarcts were significantly less common in the patients with END (4.3%, 2 of 47) than in the patients without END (24.5%, 26 of 106) (*P* = 0.003). Lower pontine infarcts were significantly more common with END than without END (63.8% vs. 42.5%, *P* = 0.015). Middle pontine infarctions were not significantly different between the two groups (*P* = 0.0659). Patients in the END group had a greater infarct diameter (*P* < 0.001) and width (*P* < 0.001) than those in the non-END group. The infarct volume was higher in patients with END than in patients without END (*P* < 0.001). There were no significant differences in the distance of the lesion from the midline of the pons in the two groups (*p* > 0.05) (Table 2).

**Table 2:** Stroke subtypes and imaging parameters with and without END in acute isolated pontine infarction

	END (*n* = 47)	Non-END (*n* = 106)	P
Stroke subtypes			< 0.0001
BABD	38 (80.9)	48 (45.3)	
SAD	9 (19.1)	58 (54.7)	
Topographic locations			
Upper	2 (4.3)	26 (24.5)	0.003
Middle	15 (31.9)	39 (36.8)	0.659
Lower	30 (63.8)	45 (42.5)	0.015
The maximum diameter, mm	13.6 (12.2, 17.8)	11.5 (8.4, 15.35)	< 0.001
The maximum width, mm	8.5 (7.4, 9.4)	6.6 (4.7, 7.7)	< 0.001
Infarct volume, mL	351.5 (259.6, 551.3)	217.4 (126.7, 338.5)	< 0.001
Lesion distance from midline, mm	2.1 (1.5, 3.6)	3.4 (1.9, 4.8)	0.013

*BABD* Basilar artery branch disease; *SAD* Small artery disease

When the factors associated with END in univariate analyses (*P* < 0.10) were entered into multivariate logistic regression analysis (adjusted for arrival time, HDL, creatinine, stroke subtype, topographic location, infarct volume, and the distance of the lesion from the midline of the pons), the results showed that infarct volume (aOR, 1.003; 95% CI, 1.001–1.005; *P* = 0.002) and BABD (aOR, 3.388; 95% CI, 1.102–10.417; *P* = 0.033) were associated with END (Table 3).

**Table 3:** Multivariate logistic regression analysis of predictors of END in acute isolated pontine infarction

Risk factor	OR	95% CI	P
Arrival time	0.983	0.965–1.001	0.065
HDL	0.416	0.114–1.520	0.185
Creatinine	0.985	0.963–1.008	0.195
BABD	3.388	1.102–10.417	**0.033**
Upper lesion	0.201	0.034–1.168	0.074
Lower lesion	1.851	0.769–4.457	0.170
Infarct volume, mL	1.003	1.001–1.005	**0.002**
Lesion distance from midline, mm	0.084	1.088–0.837	0.529

*HDL* High-density lipoprotein, *BABD* Basilar artery branch disease

END symptoms mainly include alterations in facial movements (4.2%, 2 of 47), motor function (arm) (76.6%, 36 of 47), motor function (leg) (87.2%, 41of 47), sensations (2.1%,1of 47), and language ability (19.1%, 9 of 47) (Table 4).

**Table 4:** The symptoms of Neurological deterioration and number of patients in END group

	END group
Consciousness, n (%)	0 (0)
Gaze, n (%)	0 (0)
Visual fields, n (%)	0 (0)
Facial movement, n (%)	2 (4.2)
Motor function(arm), n (%)	36 (76.6)
Motor function(leg), n (%)	41 (87.2)
Limb ataxia, n (%)	0 (0)
Sensory, n (%)	1 (2.1)
Language, n (%)	9 (19.1)
Articulation, n (%)	0 (0)
Extinction or inattention, n (%)	0 (0)

**Fig. 4:**
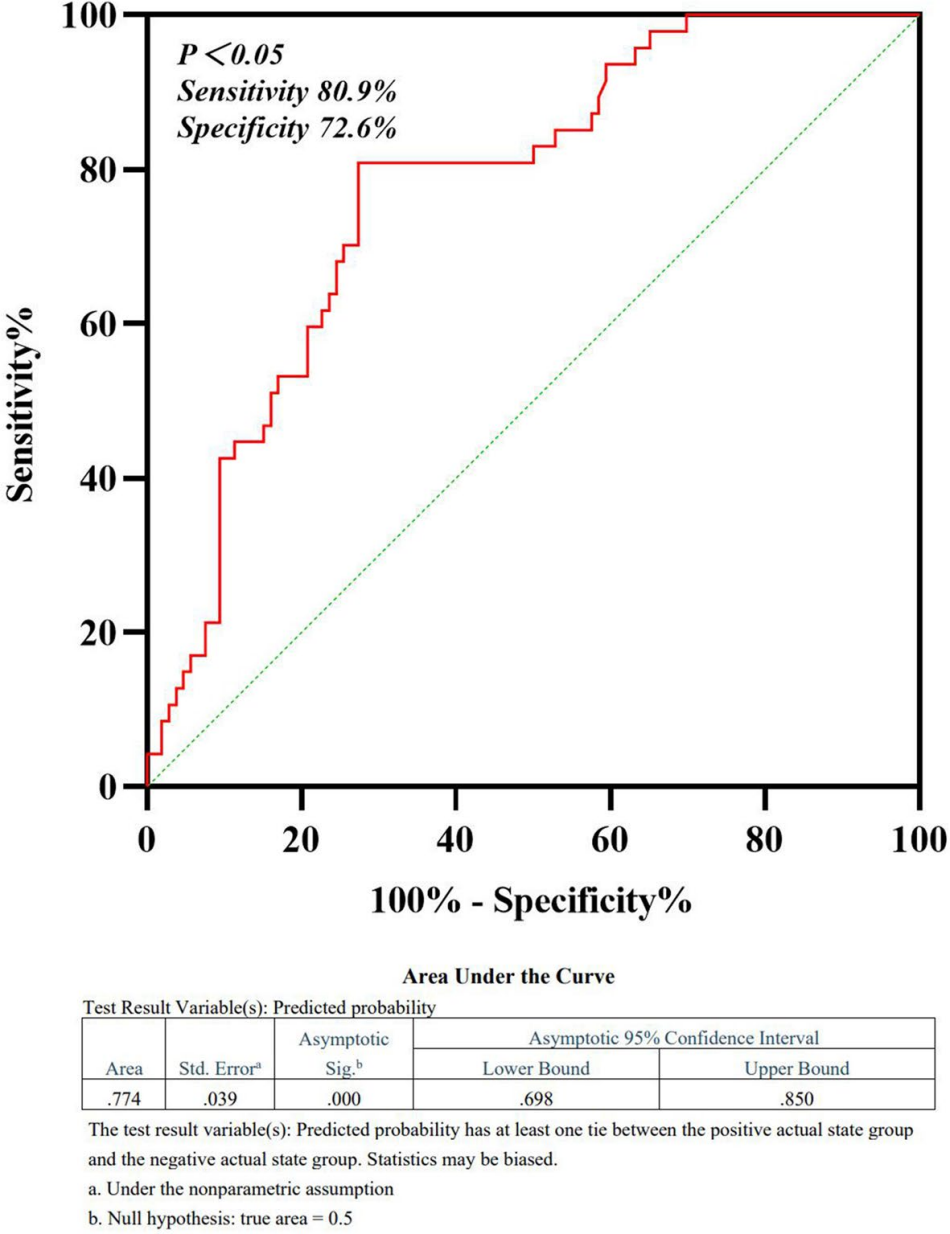
Combined ROC curve analysis of the infarct volume and BABD

The combined diagnostic value of the infarct volume and BABD was a sensitivity of 80.9%, a specificity of 72.6%, and an AUC of 0.774 (95% CI, 0.698–0.850, *P* < 0.05) (Fig. 4).

## Discussion

In our study, markers predicting END in patients with acute isolated pontine infarction were evaluated, and infarct volume and subtype-BABD were found to be associated with END in API patients. Our study suggested that patients with BABD, especially those with a larger infarct volume, may be have higher risk of END.

Penetrating arterial infarction, particularly pontine infarction, tends to progress to END, in contrast to cerebral deep penetrating artery infarction [1, 17]. Our study showed that 28.7% of the patients with isolated pontine infarction experienced END after admission, consistent with previous research findings reporting an incidence of 27%–29% of END in patients with API [1, 18]. In 1989, Caplan initially proposed the concept of BAD and BA branch disease (BABD), which was defined as lesions extending to the ventral pontine surface in the blood supply region of the paramedian pontine artery with neither evidence of large arterial stenosis (> 50%) or occlusion nor evidence of cardiogenic embolism [19, 20]. A large retrospective study showed that BABD was the most common cause of API [15], accounting for 56.2% (86 of 153) of the patients with API included in our study, and Yamamoto et al. showed a relative frequency of approximately 40% [21]. In our study, END was significantly more frequent in patient with BABD than SAD (P < 0.0001), indicating that the BABD subtype was associated with END in patients with API, consistent with the study by Gokcal et al. [10]. However, Our study did not compare long-term outcomes between patients with BABD and SVD. Erro et al. [22] indicated that patients with BABD have a worse prognosis than patients with lacunar pontine infarctions.

Our data show that the deterioration of symptoms was related to the maximum infarct volume (*P* = 0.002). Recently, a retrospective study that included 407 patients with API by Haiyan Li et al. [9] also found that infarct size might be a predictor for neurological progression with isolated acute pontine infarction (aOR 4.580, *P* < 0.0001), which was different from our study in which the infarct size was represented by the maximal data of the ventrodorsal length multiplied by rostrocaudal thickness. However, Multiple logistic regression results showed that the OR of BABD was much higher than the OR of infarct volume in our study,So We speculated that subtype-BABD has a greater impact on the occurrence of END. Interestingly, another study [8] suggested that END was not related to the size of either infarct; however, its sample size was relatively small (*n* = 38), the expansion of ischemic lesions was not correlated with END, and the actual lesion size was not measured.

According to Huang et al. [7] and Oh et al. [3], lower pons lesions may be associated with a higher probability of progressive motor deficits in patients with isolated acute pontine infarction than those in the upper and middle pons. In our study, END was only numerically higher in patients with lower pontine infarction, but the difference in multiple logistic regression results was not statistically significant, which consistent with the conclusions reported by Gokcal et al. [10]. There were also studies reported by Li et al. [3] and Nakase et al. [8] showed that the deterioration of symptoms was not related to lower pons lesions (*P* > 0.05) [8]. In addition, our study quantitatively analyzed the relationship between the distance between the lesion and the midline of the pons and END, and the results exceeded our expectations and were negative, consistent with the findings reported by Oh et al. [3], who qualitatively divided the patients into groups with paramedian pontine infarcts and extended pontine infarcts according to the axial lesion location.

In analyzing the relationships between END and the infarct location and size in patients with API from an anatomical perspective, the corticospinal tracts exhibit a scattered distribution along the corticospinal fibers in the upper pons, located in the dorsolateral part of the pontine base at the level of the upper pons, and then converge into the anteromedial surface of the upper medulla to form compact bundles [23, 24]. Therefore, Huang et al. [7] and Oh et al. [3] proposed that the increased density of the corticospinal tracts in the lower pontine region, typically in the paramedian ventral area, results in greater damage to the corticospinal tracts. Infarcts in the lower pons lesions could damage more corticospinal tracts and are prone to END. Unfortunately, Our study only found that END was only numerically higher in patients with lower pontine infarction. The anatomic view also considers corticobulbar tracts in the upper areas of the pons to be more widespread than in the lower areas of the pons and they are located most medially [25]. Therefore, the infarct size might reflect the degree of damage to the conduction tract to the greatest extent. Patients with larger infarct volumes may have more damage to corticospinal tracts and corticobulbar tracts. Damage to the conduction tract is more serious in patients with END than in those without END. We hope that more high-quality studies will be designed to study the effects of the infarct site and infarct size on END in the future.

Previous BAD-related studies have shown that early intravenous thrombolysis does not prevent the occurrence of END [13, 26, 27]. Strategies to prevent the occurrence of END are currently an important topic. Several recent studies indicated that early intensive antiplatelet or anticoagulation therapy may reduce the risk of END and improve the clinical prognosis of patients [11, 28–31]. The study by Liu et al. with a small sample size (*n* = 17) showed that early administration of tirofiban after intravenous thrombolysis with urokinase reduced the incidence of END within 3 days from onset in patients with BAD (*P* = 0.004) [31]. Recently, our observational study showed that short-term application of dual antiplatelet therapy plus argatroban seemed safe and effective at preventing END in patients with BAD [11]. Whether this reinforced anti-embolization strategy is effective in patients with API at high risk of END needs to be confirmed.

This study has the following limitations. First, it was a single-center retrospective study with a modest sample size. Second, repeat MRI was not performed after deterioration to determine whether there was infarct volume expansion in END patients and to identify the cause of the neurological deterioration. Third, our study did not compare the long-term functional outcomes of the two groups of patients.

## Conclusion

Our results indicate that the stroke subtype BABD and the larger infarct volume,which have more damage to conduction tract, are associated with END in patients with isolated API and may be useful prognostic factors for neurological progression.. Therefore, when treating patients with BABD, particularly patients with larger infarct volume, physicians must be aware of the risk of END.

## Data Availability

All original data can be obtained via email correspondence to 694,421,243@qq.com, and all charts in this study are presented in the article.

## Abbreviations

API: Acute pontine infarction
END: Early neurological deterioration
NIHSS: National institutes of health stroke scale
BABD: Basilar artery branch disease
ALT: Aminotransferase
AST: Aspartate aminotransferase
LDL: Low-density lipoprotein
HDL: High-density lipoprotein
CTA: Computed tomography angiography
MRA: Magnetic resonance angiography
DWI: Diffusion-weighted imaging
SAD: Small artery disease
SD: Standard deviation
IQR: Interquartile range
ROC: Receiver operating characteristic
